# Mechanisms of immunosenescence

**DOI:** 10.1186/1742-4933-6-10

**Published:** 2009-07-22

**Authors:** Calogero Caruso, Silvio Buffa, Giuseppina Candore, Giuseppina Colonna-Romano, Deborah Dunn-Walters, David Kipling, Graham Pawelec

**Affiliations:** 1 Immunosenescence Unit, Department of Pathobiology and Biomedical Methodologies, University of Palermo, Italy; 2 Department of Immunobiology, King's College London, UK; 3 Department of Pathology, Cardiff University, UK; 4 Center for Medical Research, University of Tübingen Medical School, Germany

## Abstract

On April 7,8, 2009 a Symposium entitled "Pathophysiology of Successful and Unsuccessful Ageing" took place in Palermo, Italy. Here, the lectures of G. Pawelec, D. Dunn-Walters and. G. Colonna-Romano on T and B immunosenescence are summarized. In the elderly, many alterations of both innate and acquired immunity have been described. Alterations to the immune system in the older person are generally viewed as a deterioration of immunity, leading to the use of the catch-all term immunosenescence. Indeed, many immunological parameters are often markedly different in elderly compared to young people, and some, mostly circumstantial, evidence suggests that retained function of both innate and acquired immunity in the elderly is correlated with health status. What is often not clear from studies is how far immune dysfunction is a cause or an effect. A better understanding of immunosenescence and mechanisms responsible for proven deleterious changes is needed to maintain a healthy state in later life and to design possible therapeutic interventions.

## Background

The immune system of older people is usually perceived as declining in fidelity and efficiency with age, resulting in an increased susceptibility to infectious diseases and pathological conditions relating to inflammation (e.g. cardiovascular disease, Alzheimer's disease) or autoreactivity (e.g. rheumatoid arthritis). This overall change in immunity is loosely termed "immunosenescence". The individual contributing factors to immunosenescence are many and varied, due to the multi-factorial complexity of the immune system. It is often difficult to determine whether changes in a particular cell type are intrinsic to that cell, or caused by environmental changes, or both. This is particularly the case for lymphocytes, where the interplay between B cells and T cells is crucial for effective responses, so if one subset is affected it will change the function of the other one [1-3].

## Impact of Cytomegalovirus (CMV) infection on immunosenescence

Anecdotally, the clinical relevance of immunosenescence is well-documented, but exact detailed information is, in fact, hard to come by. Immunosenescence is a very vaguely-defined descriptive term covering the deleterious age-associated changes to immunity observed in all mammals studied so far. As immunity almost certainly evolved to protect against infectious disease, which is a major cause of reduced lifespan, correlations between immune function and longevity have been sought for many years. Early studies indicated that responses to mitogens predicted mortality to some degree, and since then many studies have probed associations between survival and parameters of both innate and acquired immunity. An emerging consensus suggests that maintenance of appropriate immunity is essential for exceptional longevity and, by implication, also for "normal" longevity [4]. While all components of innate and acquired immunity are changed with age, the clinical impact of these changes is not clear, and mechanisms of and markers for immunosenescence are controversial. In humans, cross-sectional study design raises many difficulties – potentially confounding the interpretation of the published data. Examining immune status in the current elderly is rather like an astronomer examining the far-away cosmos: we are seeing the results of events that happened a long time ago, when circumstances were different from those applicable nowadays. These differences, which cannot be controlled for, include genetics, environment, nutrition, developmental variables and pathogen load [5]. The advantages and disadvantages of longitudinal studies on the same individuals emerged in the context of the pioneering OCTO/NONA studies of people >85 yr of age, which resulted in the definition of an "immune risk profile" (IRP) [5-8]. Although this is a concept which has become increasingly accepted of late, it should be emphasized that the IRP has only been shown to predict mortality in very elderly Swedes, on the basis of very limited data. These studies must be repeated and performed in other populations too. On the basis of even less data, we can say that it seems that the IRP is not predictive of excess mortality at 55 yr baseline, but might start to become so at 65 yr (on 10-yr follow-up). Because one very strong influence on the IRP is infection with CMV, it will be extremely important to test whether immune signatures like the IRP are informative under other circumstances, in different populations, and whether polypathogenicity has an additive effect. There is some epidemiological evidence for excess mortality in CMV-positive populations, which is further increased in those co-infected with hepatitis A and B as well. There is also some emerging evidence that CMV antibody titer may also be informative in this regard: individuals in the upper quartile had significantly reduced survival times compared to those in the lower quartile. The marked influence of CMV on immune signatures is illustrated in the finding that cross-sectional studies on several different European populations clearly indicate that the consensus view of T cell immunosenescence (that the fraction of naïve CD8 cells decreases in the elderly and the fraction of late-differentiated memory cells increases) does indeed hold true – but only for people who are infected with CMV. Such individuals also have higher levels of C-reactive protein, indicating that they are more likely to suffer "inflammaging", itself linked with increased occurrence of diabetes and other inflammatory diseases, as well as general frailty and increased mortality. This too may therefore be markedly influenced by CMV. Infection with other persistent herpesviruses, at least EBV, HSV and VSV, does not appear to have any similar effect [5,9]. The uniqueness of CMV in this context remains enigmatic. We propose that there may have been some advantage in early life to being CMV-positive – possibly precisely because of the enhanced pro-inflammatory status in infected people which might have had a protective effect against infection with other pathogens under conditions in the wild. It is thus concluded that immune signatures are indeed informative for "immunosenescence", which predicts mortality, but that these immune signatures are materially influenced by CMV infection [10]. Any immunogerontological study must therefore take CMV status into account. Although immunosenescence is clearly not caused by CMV, if only because not all elderly people are CMV-positive, this infectious agent seems to have a large impact on immune parameters in later life and may contribute to increased morbidity and eventual mortality. If truly carrying a benefit in early life, this would be yet another example of "antagonistic pleiotropy" which seems almost to constitute one of the few general laws of ageing [11].

## B immunosenescence

Literature on immunosenescence has focused mainly on T cell impairment, but the B cell compartment is also affected in aged. The quality of the antibody response is substantially impaired. Until recently it was considered that the most likely cause of B cell failure was a lack of effective T cell help in a T-dependent reaction. Thymic involution is well known, and there is a substantial literature on the functional decline of T cells with age. However, there are T-independent functions of B cells, such as the polysaccharide responses that are crucial for anti-bacterial protection, which also appear to be lacking in later life. Additionally, there is emerging evidence to suggest that B cells are important antigen presenting cells in their own right and can be key regulators of T cell development, leading us to speculate that some of the failures of T cell function may yet be blamed on insufficient help from B cells! Changes in B cell number and repertoire have been described, and decreased IgM and IgD levels in the elderly suggest a shift from the naïve (CD27-) compartment of the B cell branch towards the memory (CD27+) compartment. However, these data are controversial since not all studies have shown this [12-14].

Circulating B cells can be divided on the basis of their expression of IgD and CD27 into different functional subsets. In the aged, a double-negative (DN) IgD-CD27- B cell subset is significantly increased [14]. Most of these cells are IgG^+^. Preliminary data on telomere length and expression of the ABCB1 transporter and anti-apoptotic molecule, Bcl2, suggest that DN cells have the markers of memory B cells. Furthermore, these cells do not seem to act as antigen presenting cells, nor do they express significant levels of the CD40 molecule necessary to interact with T lymphocytes through the ligand, CD154. Hence, these expanded cells may be late memory or exhausted cells that have down-modulated the expression of CD27 and filled the immunologic space in the elderly. These cells might be the age-related manifestation of time-enduring stimulation or dysregulation of the immune system [14]. Interestingly this DN B cell population is increased also in patients affected by Lupus [15] and in healthy subjects challenged with respiratory syncitial virus (RSV) [16].

Of interest, B naïve lymphocytes are increased in the offspring of healthy centenarians [17]. It is well known that older offspring of centenarians, who are in their 70 s and 80 s, have a survival advantage when compared with control subjects of the same age range whose parents died at an average life expectancy [18]. The main lymphocyte differences observed between the two groups concern B cells. Indeed naïve B cells are more abundant in centenarian offspring. These data are similar to that found in previous studies on younger subjects. So, the B cell compartment of the older offspring of centenarians seems to have more in common with that of younger controls than with control subjects of a similar age [17].

## Human B cell repertoire diversity in old age

It is clear that the humoral immune response to challenge is impaired in later life, since the titre and affinity of antibodies raised by vaccination are consistently lower for a variety of different vaccine challenges [19]. The actual number of B cells does not appear to change with age in proportion to the decrease in vaccine efficiency. In fact, in mice, there is little evidence for any change in overall B cell numbers. Yet there are reports of perturbations in the germinal centre (GC) reaction, which is key to the development of effective B cells in a T-dependent response. Some groups studying mice have reported a decrease in the size and number of GCs responding to challenge [20]. Other studies in humans have not shown any difference in GC number [21], but have shown more subtle differences in the dynamics of the response within the individual GCs [22]. The strength of selection in the affinity maturation process within the germinal centre was shown to decrease with age in the germinal centres of Peyer's patches in the gut. This was not seen in those of the spleen, so there are tissue-specific differences occurring that are not always easy to elucidate in humans [22]. A decrease in function of GCs in the gut could be due to a number of factors. Availability of T cell help is an obvious one, although we did not see any differences in the numbers of CD4+ T cells present in these follicles [21].

Diversity of the available pool of B cells is another possible factor. It has been shown in mice that the lymphoid lineage is decreased at the expense of the myeloid lineage and the bone marrow B cell output decreases with age [23]. Although this has not been shown in humans it is a strong possibility. A decreased output of naïve B cells, coupled with the accumulation of memory cells from previous immune challenges along the lifecourse, may well result in a reduction of B cell diversity. This would be especially true in the mucosal immune system where the antigenic challenge is frequent. Loss of diversity in a population of B cells would theoretically impair the B cell response to new challenges as the available repertoire from which to find an effective responder would be decreased. This would be particularly important if, as suggested, a larger proportion of the population are memory cells that had already been through the affinity maturation process [14]. Their B cell receptors would be more specific for a particular antigen and may therefore have lost the flexibility of their antigen binding site that might have otherwise allowed them to accommodate a broader range of antigens. B cell diversity in the peripheral blood of participants from the Swedish "NONA" longitudinal study using a method of B cell spectratyping was recently explored. This takes advantage of the fact that the CDR3 region of the immunoglobulin heavy chain is extremely diverse, so that when the region is PCR amplified the resultant fragment sizes follow a Gaussian distribution, providing that the starting material contains over 300 B cells. A change in diversity of the population can be detected by deviation from the normal distribution. Most spectratypes of a control group aged under 50 years show little variation. However, about a third of the older group, aged 86 to 94, has significant deviation from normal, indicating a loss of diversity of the B cell population in the peripheral blood. This loss of diversity correlated strongly with the health status of the individual, a loss of diversity being associated with those classified as "frail" [24]. Further studies will have to be undertaken to determine the exact relationship between loss of diversity and frailty, and whether the loss of B cell diversity substantially affects the response to antigen challenge. However it is becoming clearer that the B cell population is substantially altered in old age and that this has a significant contribution to immunosenescence.

## Conclusion

In the elderly, many alterations of both innate and acquired immunity have been described. These alterations are generally viewed as a deterioration of immunity, leading to the use of the term immunosenescence. This process is also characterized by chronic inflammatory status. Hence, immunosenescence is responsible for the increased susceptibility of elderly to infectious diseases as well as being at the root of the biological mechanisms responsible for inflammatory age-related diseases [1-4]. A long life in a healthy, vigorous, youthful body has always been one of humanity's greatest dreams. Hence a better understanding of immunosenescence, and the development of new strategies to counteract it, are essential, not only for anti-ageing strategies aiming at rejuvenation, but, more importantly, with the aim of prolonging healthy life by preventing infectious diseases and thereby improving the quality of life in later years [25,26].

## Competing interests

The authors declare that they have no competing interests.

## Authors' contributions

All the Authors drafted the manuscript and approved the final manuscript.
